# An Inexpensive Suture Practice Board

**Published:** 2015-12-08

**Authors:** Dexter Weeks, Morton L. Kasdan, Bradon J. Wilhelmi

**Affiliations:** ^a^University of Louisville School of Medicine, Louisville, KY; ^b^Robley Rex Veterans Affairs Medical Center, Louisville, KY; ^c^Department of Surgery, Division of Plastic Surgery, University of Louisville, Louisville, KY

**Keywords:** suture practice, suture board, inexpensive suture practice, suturing technique, suturing instruction

## Abstract

**Objective:** We provide a design for an effective suture practice board for surgical instruction that is both easily assembled and repaired. **Methods:** This model's design is achieved through inexpensive materials that do not compromise adequate simulation through repetitive use. We used a wooden board, synthetic microfiber cloth, and metal plates and screws to create the suture board. Two pieces of synthetic microfiber cloth, folded along the long axis, were attached to the outer edges of the wooden board using an electric screwdriver, with the metal plates and screws to secure the attachment. **Results:** Upon completion of construction, we have a board sufficient for instructing various suturing techniques. **Conclusions:** Our suture board design provides an effective practice material that is an improvement in cost, as well as reusability compared with other models. Our board has the advantage over animal tissues, such as chicken's or pigs’ feet, because it is not perishable and maintains its durability over extended periods of time. This model is advantageous compared with other commercially available synthetic models because the materials are cheaper and more easily replaced. Our suture board model provides sufficient simulation to enhance the user's skills across various suturing techniques in a manner that is cost-effective in production and maintenance.

Proficiency in suturing is essential for maintaining the postsurgical function of the tissue as well as improving aesthetics of the healed wound. Effective instruction in suturing techniques outside of the operating room requires access to practical tools designed for educational consistency and adequate repetition.^1^ Aside from the surgical instruments, the users should have a suture surface that is durable and easily replaceable. With this purpose in mind, we have designed a simple, yet effective suturing practice board that provides many benefits over traditional and commercial practice materials.

## METHODS

**Figure 1:**
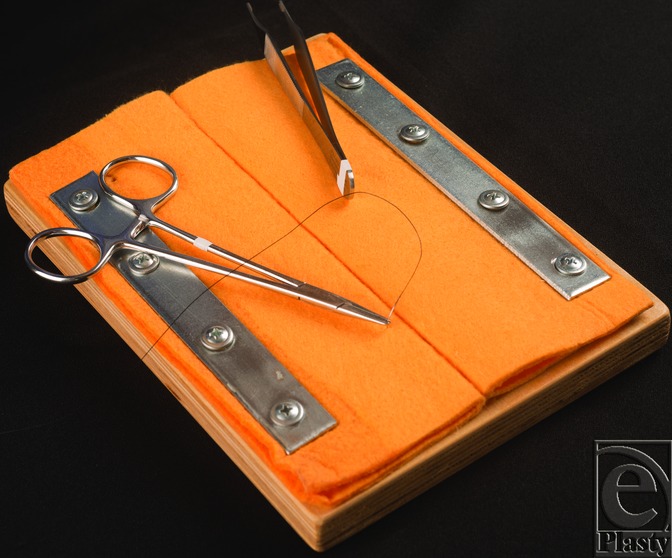
A suture board after completed construction.

To begin construction of the board, we used a piece of wood with dimensions 18 cm long by 15 cm wide by 2.54 cm thick as the base. Second, we cut 2 pieces of synthetic microfiber cloth with dimensions 18 cm by 14 cm. Both pieces of the cloth were folded along the long axis and placed on the board so that the folded edge of each cloth faced one another. Each cloth was placed within proximity to simulate proper wound closure upon suturing. Next, each cloth was secured to the wood board at the outer edges with plates and screws (Fig 1).

## RESULTS

This model tolerates passage of tapered and cutting needles of varying diameters with regular use for months. The properties of the synthetic microfiber cloth allow it to be manipulated into eversion or inversion. This model is designed so that the part of the cloth that represents the wound edge provides structure that simulates the dermal-epidermal junction. The folded edge allows adequate simulation of buried and superficial stitches that the user can easily visualize.

## DISCUSSION

**Figure 2:**
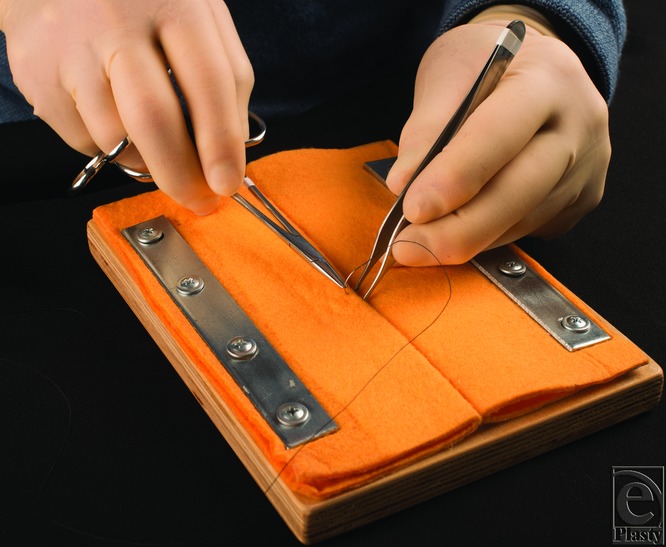
A user passing a needle through the suture board.

Our technique for designing this suture board provides simple assembly, cost-efficiency, and practical benefits not seen with other models for suturing practice.^2^ The practicality of this model is justified through the ease in replacing the materials that comprise the device. Commercially available suture models range from $16 to $210. Our model is cheaper to produce initially and maintain for a long term. This model costs $6 to create. The wooden board is free scrap wood obtained from woodworking supply stores, but commercially available wood boards cost $4. The 2 mending plates and wood screws cost $2.50, and the microfiber cloth costs $3.50. Commercial models must be repurchased once expired, but our model requires only replacement of the microfiber cloth. A particularly salient advantage of this model is the durability over long periods of time compared with traditional suturing practice materials. These include tissues such as pig feet, chicken feet, and banana peels. Traditional models confer tactile similarity to human skin that is progressively diminished with the continued use due to their perishable nature. Frequent replacement of perishable tissue for long-term practice also reinforces that our model is more economical. The microfiber cloth in our model has pliability for eversion not matched by commercial boards. The pliability and capability for eversion or inversion are essential to show when the user practices proper superficial closure. Commercial models with synthetic polymers are often created using a single sheet of material with a central incision to serve as the wound. These models allow the user to practice approximating tissue but fail to simulate eversion. Our model's wound edge is configured to show when the user passes needles at proper depth for deep dermal or running subcuticular sutures. Many commercial models lack design allowing buried suture practice. These boards either are created with total thickness unaccommodating for passing needles deeply or have shallow wounds. Those that match our model's capacity for deep suturing are at least 4 times greater in price. The ability of our model to tolerate tapered and cutting needles with varying diameters makes it sufficient for regular instruction and practice of suturing technique (Fig 2).

## CONCLUSION

Pertinent to the development of proficient suturing technique is the availability of quality practice materials. This model of suture board meets and exceeds the necessary requirements for effective practice, entailing both sufficient simulation of primary approximation on human tissue and reliability of materials through multiple uses.
